# Early olfactory pre-conditioning during sensitive developmental periods is associated with enhanced detection performance in working dogs

**DOI:** 10.1007/s11259-026-11237-w

**Published:** 2026-05-14

**Authors:** Gloria Durán-Arroyo, Isabel Cuadrado-Gordillo, David Alhadeff-Von Bormann, M. J. Fernández, Edvil Josué Pichilingue-Chalco, Dominique Grandjean

**Affiliations:** 1 Universidad de Extremadura, Badajoz, España; 2 Facultad de Educación y Psicología, Universidad de Extremadura, Badajoz, España; 3 Tactical k9 Training, Madrid, España; 4 Hospital Universitario Infanta Cristina, Badajoz, España; 5 Facultad de Psicología, Universidad Autónoma de Madrid, Madrid, Spain; 6 Institut NOSSAÏS. École Nationale Vétérinaire d’Alfort (EnvA), Maisons-Alfort, France

**Keywords:** Dogs, Explosive agents, Odor detection, Olfaction, Animal training, Behavior

## Abstract

Early olfactory imprinting has been proposed as a strategy to enhance the performance of detection dogs, yet empirical evidence from operational contexts remains limited. This study evaluated the effects of early exposure to target odours on four core detection variables: sensitivity under tight-containment conditions, reaction distance, work endurance, and detection reliability. Twenty operational adult dogs served as controls, and four dogs underwent an early-learning protocol beginning during sensitive stages of neurosensory development. All dogs completed parallel test batteries under equivalent conditions, and data were analysed using Welch’s t-tests with effect size estimation. Dogs exposed to early imprinting showed higher detection sensitivity in tight-containment conditions and reacted to the target odour at substantially greater distances than control dogs. Work endurance was also higher in early-learning dogs, which maintained effective performance for extended periods compared to conventionally trained dogs. In contrast, false-positive behaviour did not differ between groups, indicating that response inhibition and decision control were not modified by early imprinting alone. Taken together, the findings indicate that early olfactory imprinting is associated with improvements in detection sensitivity, reaction distance, and work endurance, while detection reliability remains unchanged. These results suggest potential benefits of integrating early imprinting protocols into breeding and training programmes for detection dogs and highlight the need for larger, controlled studies to further characterise their developmental and practical implications.

**Supplementary Information:**

The online version contains supplementary material available at 10.1007/s11259-026-11237-w.

## Introduction

The relationship between humans and dogs has evolved from domestication to a multifunctional bond that includes operational collaboration in security, detection, rescue, and therapeutic assistance. This expansion is supported by the dog’s exceptional sensory abilities, particularly its olfactory capacity, estimated to be between 10,000 and 100,000 times greater than that of humans, allowing the detection of chemical compounds at extremely low concentrations (Oliveira et al. 2012). Dogs can discriminate over a million odors, while humans rarely exceed 4,000 (Allen and Bekoff 1999).

Comparative studies show that untrained dogs often rely on visual or kinesthetic strategies when searching, whereas trained detection dogs consistently employ olfaction as their primary modality (Smith et al. 2003). Thus, training modulates the functional expression of innate sensory abilities.

Understanding early development in puppies is central to optimizing detection performance. Early learning and imprinting, initially described by Lorenz, shape future behavioural patterns (Kalikow 1983). Although imprinting was first identified in birds, research shows that it also affects mammalian species, including canids (Beach and Jaynes 1956; Dietz et al. 2018; Wright and Russell 1983). The imprinting period in dogs extends through the first four months of life (Kretchmer and Fox 1975), and experiences in this window significantly influence social, aggressive, reproductive, and territorial behaviours (Johnson et al. 1985).

Some authors propose that these “critical periods” are better understood as flexible “sensitive periods” influenced by environment and interventions (Bateson 1979, Parker and Mellor 2011). This opens possibilities for structured learning beyond early infancy.

During the first 30 days of life, puppies are born blind, deaf, and essentially anosmic, but display functional tactile, thermal, and gustatory responses that facilitate survival (Navarrete 2004; Lorenz 1950). Around days 20–25, auditory function emerges alongside activation of the temporal cortex and the onset of attachment behaviours (Vaz Ferreira 1984). Socialisation and olfactory recognition intensify between weeks 3 and 12, overlapping with the most sensitive phase of associative learning (Lorenz 1950; Ackerman 1997).

Early separation from the mother before week 7 has been linked to behavioural disorders and deficits in social functioning (Hafez ESE, 1968; Slabbert and Rasa 1993; Ferrari 2001). Maternal care, sibling interactions, and exposure to novel stimuli shape communication and hierarchy mechanisms foundational for adult performance (Sire 1968; Gazit and Terkel 2003).

The Von Bormann protocol aims to capitalise on early behavioural plasticity to induce functional olfactory learning during the imprinting period (Ortiz-Leal et al. 2020). According to Sire (Sire 1968), reciprocal social interaction enables the dog to perceive the human as a partner, a key element in guide–dog cooperation (Sarría-Echegaray et al. 2014). Early structured training aligned with motivational principles reinforces this cooperation (Sire 1968; Gazit and Terkel 2003).

Despite evidence supporting early learning, few empirical studies have examined its specific impact on olfactory detection capabilities (Parker and Mellor 2011; Hall et al. 2014; Hall et al. 2016). This study contributes to that gap by evaluating early socialisation, olfactory imprinting, and structured stimulation in working puppies.

From a developmental standpoint, dogs can encode chemosensory information extremely early. Experimental evidence in domestic dogs indicates that perinatal exposure to odorants can shape later odor preferences, supporting the biological plausibility of very early odor learning (Wells and Hepper 2006; Battaglia 2009). In mammalian models, neonatal olfactory learning has been linked to sensitive-period mechanisms and distinct early-life circuitry supporting durable changes in odor-guided behavior (Moriceau and Sullivan 2004; Sullivan and Holman 2010). Importantly, the present protocol also fits a learning-theory framework: pairing target odors with appetitive outcomes constitutes classical conditioning, and controlled studies in dogs have shown that odor pre-exposure and odor–reward pairing can facilitate later odor discrimination learning and increase detection sensitivity (Furton et al. 2015; Polgár et al. 2015).

Accordingly, the main objective of this study was to evaluate whether structured early-life exposure to target odors during the imprinting/sensitive-period window yields measurable advantages in adult detection performance relative to conventional training.

## Methodology

### Study design and setting

This study used a longitudinal observational comparative design to evaluate whether very early olfactory learning during sensitive developmental periods is associated with later differences in operational explosive-detection performance. Group allocation was non-randomised and determined by training history (early-learning exposure vs. conventional training). The unit of analysis was the individual dog.

All procedures described correspond to routine training and operational evaluation practices implemented in real working-dog contexts. No invasive procedures were performed.

### Animals and groups

#### Early-learning group

The early-learning group comprised four dogs with complete, systematically recorded developmental histories:

- Hope (female; Belgian Malinois): neonatal imprinting initiated at 7 days of age (hand-reared).
- Angus (male; Chesapeake Bay Retriever × Labrador mix): early-learning initiated at approximately 21 days of age.
- Dora (female; littermate of Angus; same cross): early-learning initiated at approximately 21 days of age.
- Vaiana (female; Malinois × mixed Chesapeake-type cross): early-learning initiated at approximately 20 days of age.

#### Control group

The control group included 20 adult operational explosive-detection dogs trained using conventional methodologies:

- Operational age range at evaluation: 1–5 years (fully operational stage).
- Typical age at onset of conventional training: 9–12 months.
- Discipline: explosives detection.
- Origin: operational canine units from State Security Forces and professional security companies.

(*Note: individual-level breed/sex distribution for controls was not recorded in a standardised table in the available dataset and is therefore not reported here.*)

#### Early-learning protocol

The early-learning intervention consisted of structured olfactory exposure paired with reinforcement, progressively adapted to developmental stage, and implemented across three phases. The protocol is referred to in this manuscript as an early olfactory imprinting protocol (hereafter, “Von Bormann protocol” for internal consistency with prior documentation).

#### Phase 1 — early stimulation in Zephir’s litter (starting ~ 21 days)

Two puppies from Zephir’s litter (Angus and Dora) later contributed complete performance data. Puppies were introduced to olfactory-guided exploration using food as the primary reinforcer.

**Fig. 1:**
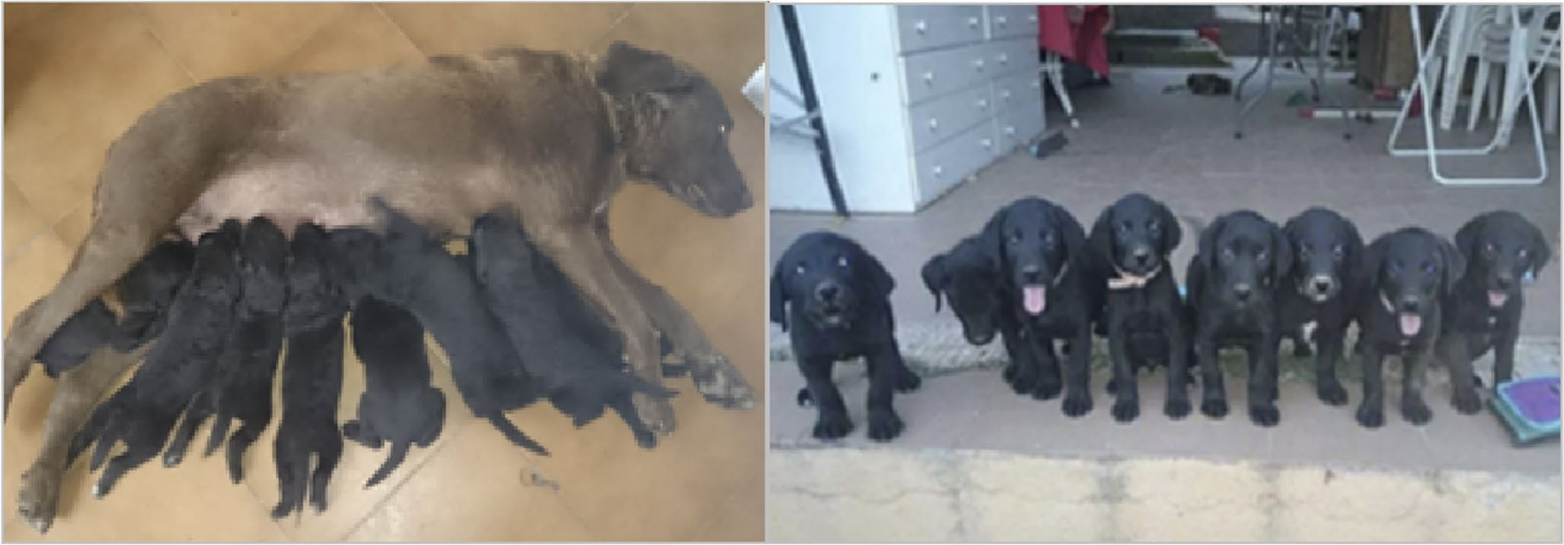
Early developmental context of Phase 1. Zephir with her litter at approximately five weeks of age, illustrating the early developmental environment in which olfactory-guided free-search stimulation was initiated for selected puppies (Angus and Dora)

- Free-search induction: food was dispersed over progressively larger areas to encourage independent exploration guided by olfaction in the presence of background odours (vegetation, objects, humans, other dogs). Environmental complexity increased gradually (auditory distractors, presence of conspecific movement, changes in handler position) to reduce dependence on a single context.
- Session structure: repeated short sessions per day, aligned with normal feeding schedules; occasional individual sessions were included to promote independent problem-solving and reduce copying/following behaviours.
- Transition to conditioned marking: from approximately 2 months, a sit-marking response was shaped to obtain a toy reward, establishing the marking behaviour later used in operational detection Fig. 1.

#### Phase 2 — neonatal olfactory imprinting in hope (starting day 7)

Hope was orphaned/rejected at 7 days and hand-reared, enabling controlled, repeated odour exposure during feeding.

**Fig. 2:**
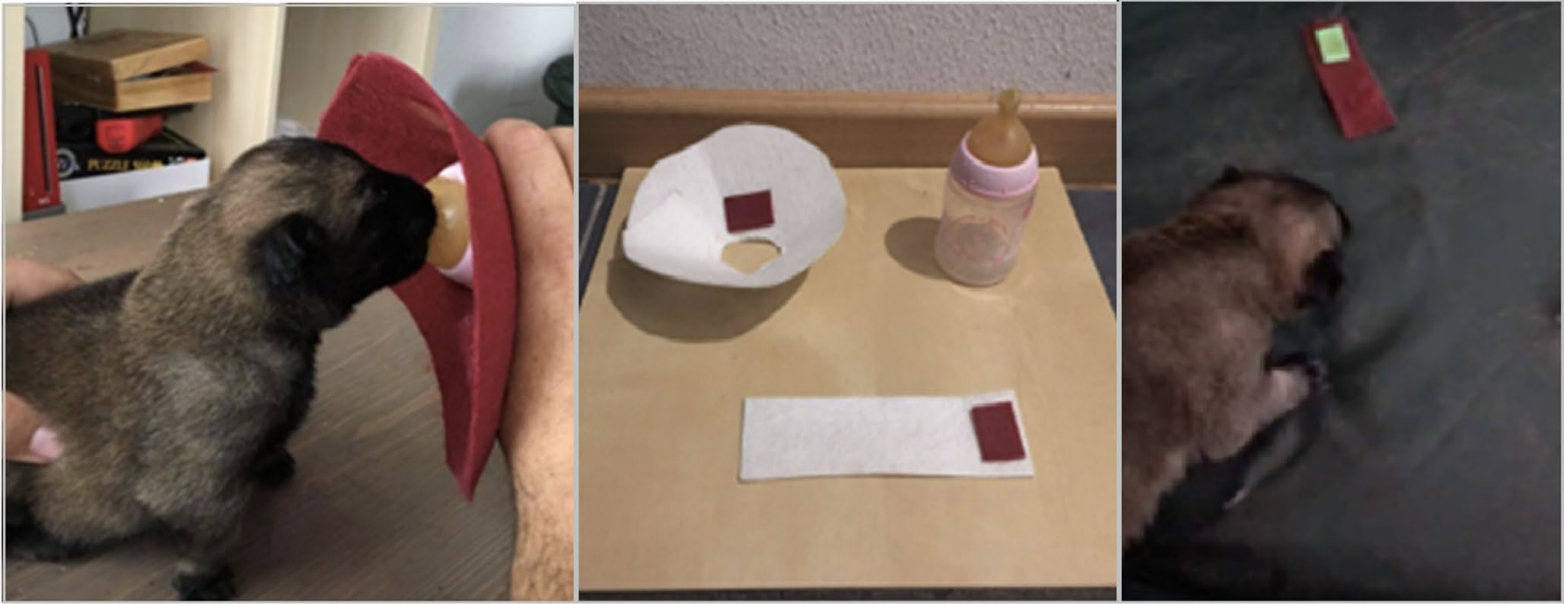
Neonatal olfactory imprinting during feeding. Hope at seven days of age during bottle feeding with a modified feeding device presenting a target explosive odour, used to establish repeated odour–reinforcer pairings during the neonatal period

- Odour–reinforcer pairing during bottle feeding: a feeding bottle was modified so that a paper strip impregnated with a target explosive odour was presented consistently during suckling episodes, generating repeated pairing of odour exposure with feeding reinforcement.
- Welfare and handling procedures: thermal support, gentle handling, and stimulation normally provided by maternal care (e.g., post-feeding cleaning and perianal stimulation when required in neonatal care) were used to maintain physiological stability and minimise distress.
- Early discrimination checks: during awake non-feeding periods, Hope was presented with (i) target-odour strips and (ii) non-impregnated control strips. Orientation/approach and increased sniffing behaviour were used as behavioural indicators of differential response (observational monitoring; no parallel scorer design).

- Introduction of multiple odours: additional explosive odours were introduced sequentially as repeated pairings during feeding episodes; later, combined exposure was implemented as development progressed as it is showed in Fig. 2.

####  Phase 2 extension — transition to search and point-to-point work (from ~21 days)

After the onset of weaning (around day 21), the protocol shifted from pairing during feeding to active search tasks.

**Fig. 3:**
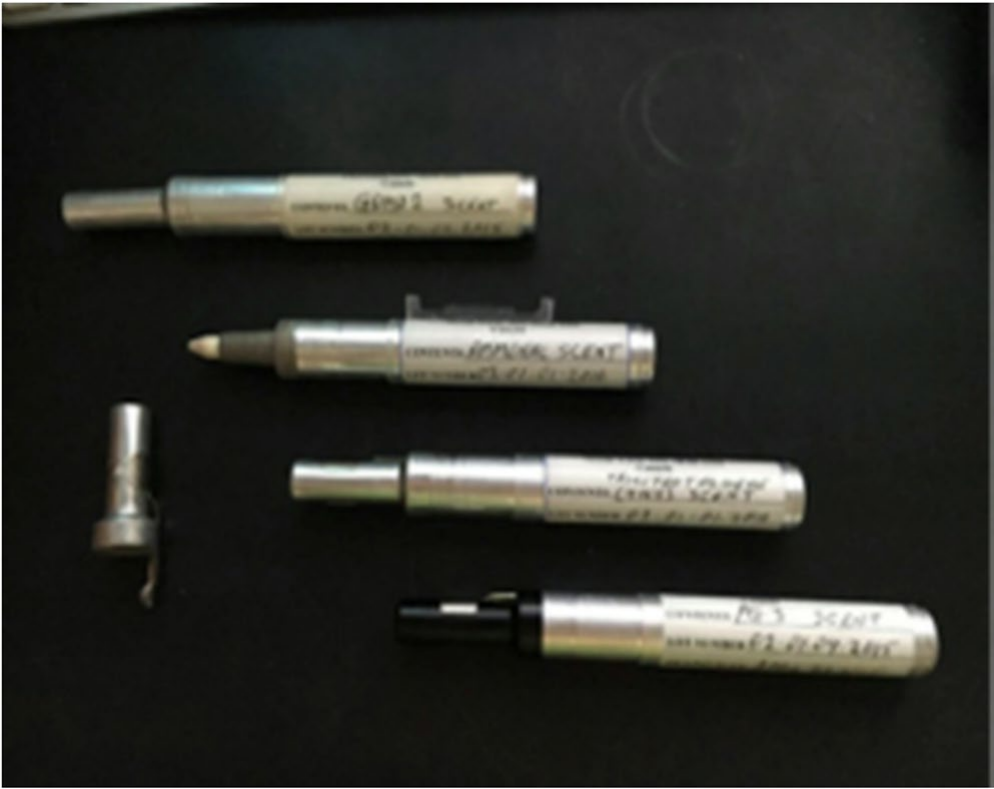
Odour stimuli used for early imprinting and training. Microtrace explosive pens containing standardised trace quantities of explosive compounds (1–7 µg/mL), employed for controlled olfactory exposure during neonatal imprinting, early search tasks, and point-to-point discrimination exercises

**Fig. 4:**
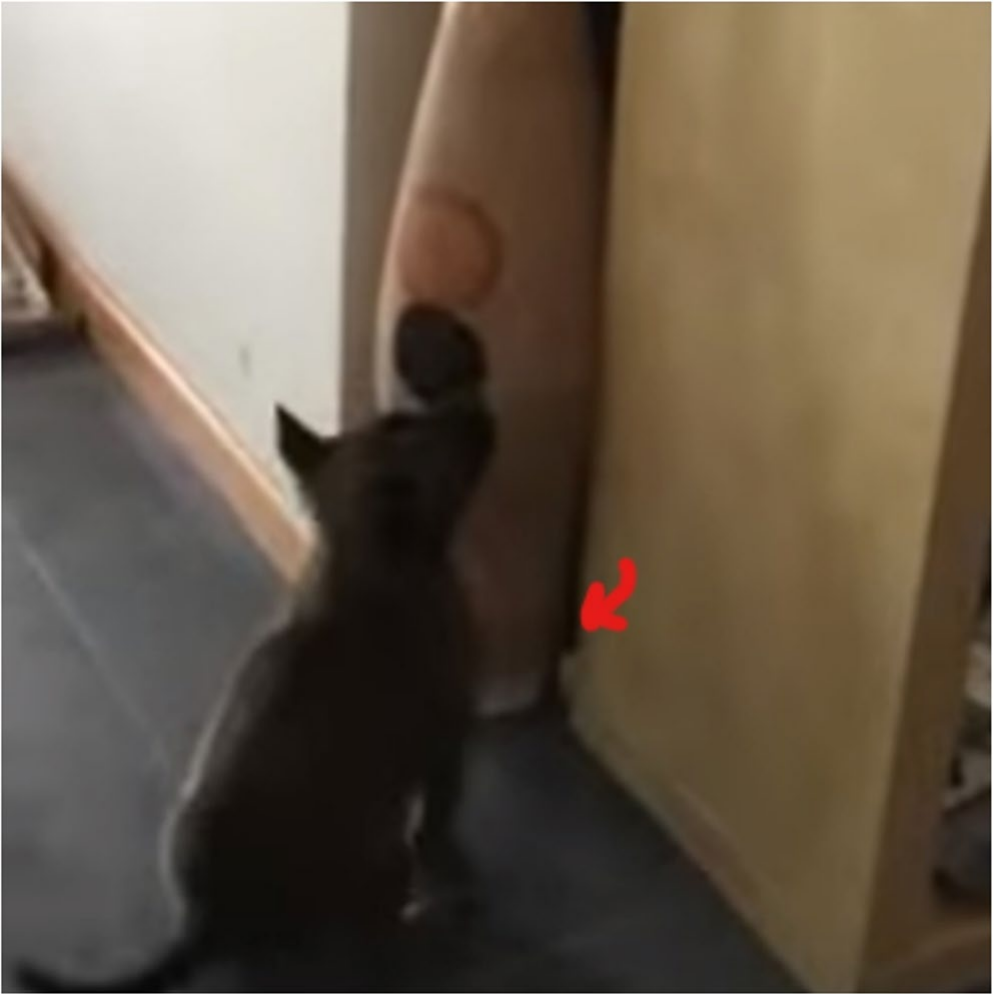
Early marking behaviour following olfactory detection. Example of a sit-marking response performed by Hope during an early search task (> 45 days of age), illustrating the conditioned indication behaviour shaped during the transition from imprinting to active search work

- Ground-search exercises: target odour samples were hidden in different locations within environments containing common background odours (food, cleaning products, other animals).
- Point-to-point discrimination: a simplified array of perforated containers/cans was used, with one containing the target odour and others containing either no odour or distractors (e.g., herbs, perfume, air fresheners, food). The puppy was guided to sample each location. Correct indication was immediately reinforced with play (toy reward), consistent with positive reinforcement training principles Figs 3 and 4.

#### Phase 3 — early developmental conditioning in vaiana (starting ~ 20 days)

Vaiana (Hope × Angus) underwent an adapted early-learning protocol starting at approximately 20 days.

- Reduced-intensity pairing: one structured odour pairing session per day with two explosive odours.
- Progression: incorporation of free-search and point-to-point exercises following the same developmental logic as Hope’s protocol.

#### Odour stimuli and training materials

Explosive odours were provided using microtrace pens containing standardised explosive traces at concentrations between 1 and 7 µg/mL. Microtrace material was applied to:

- paper strips for neonatal pairing,
- feeding-related items in early search tasks,
- target containers used in point-to-point exercises.

Materials were handled and stored following manufacturer recommendations and applicable operational safety procedures.

1. Detection sensitivity under tight-containment conditions (tightness factor).

Tightness factor quantified detection performance when the target odour was presented in a tightly sealed container designed to minimise odour leakage.

$$\begin{array}{c}Tightness\;factor\;(\%)\;=\frac{Correct\;positive\;detections}{Total\;valid\;trials\;}\times100\end{array}$$

2) Reaction distance

Reaction distance was defined as the distance (cm) at which the dog first exhibited a clear detection response while approaching the target source during search activity. The per-dog value was calculated as the mean across recorded sessions.

3) Work endurance

Work endurance measured the duration (minutes) during which the dog maintained effective operational performance without disengagement (fatigue/boredom/frustration indicators as documented in training logs). The per-dog value was computed as the mean across recorded sessions:

$$\begin{array}{c}Work\;endurance\;(min)=\frac{\sum_{i=1^{t_i}}^n}n\end{array}$$

4) Detection reliability

Detection reliability was operationalised using false-positive responding and a derived detection rate.

A false positive was defined as a full marking response (typically sit-marking) in the absence of a target sample.

$$\begin{array}{c}False-positive\;index\;(FPI)=\frac{False\;-positive\;response}{Total\;sessions}\end{array}$$

$$Detection\;rate=1-FPI$$

#### Statistical analysis

All analyses were conducted in SPSS, using the dog as the experimental unit. For each variable, per-dog values were compared between the early-learning and control groups. Due to unequal group sizes and expected heterogeneity of variance, Welch’s t-test was used. Effect sizes were estimated using Hedges’ g (appropriate for small samples). Assumptions were evaluated using Shapiro–Wilk (normality) and Levene’s test (variance homogeneity). Statistical significance was set at *p* < 0.05.

#### Ethics and welfare compliance

This study did not involve invasive procedures or experimental manipulation beyond routine training and operational evaluation. All activities adhered to animal welfare principles and standard working-dog handling practices. In line with Directive 2010/63/EU and Spanish Royal Decree 53/2013, the activities described do not constitute regulated animal experimentation requiring ethics committee approval.

## Results

A total of 24 dogs were included in the analyses: 20 conventionally trained operational explosive-detection dogs (control group) and 4 dogs exposed to early olfactory imprinting during sensitive developmental periods (early-learning group). All analyses were conducted using the individual dog as the statistical unit. Individual- and session-level data supporting the results are provided in the Supplementary Tables (S1–S4).

### Detection sensitivity under tight-containment conditions

Detection sensitivity under tight-containment conditions differed clearly between groups. Dogs exposed to early olfactory imprinting showed a substantially higher mean detection rate (87.50 ± 2.08%, *n* = 4) than conventionally trained control dogs (64.70 ± 8.74%, *n* = 20). Importantly, this difference was consistent across individuals: all early-learning dogs displayed detection rates above the upper range observed in the control group (Supplementary Table S1), indicating a robust group-level enhancement in performance under restrictive odour-leakage conditions.

### Reaction distance

Reaction distance showed the largest separation between groups. Early-learning dogs reacted to target odours at a mean distance of 901.00 ± 89.48 cm (*n* = 4), whereas control dogs reacted at markedly shorter distances (23.89 ± 3.83 cm, *n* = 20). No overlap was observed between individual values from the two groups (Supplementary Table S2). These results indicate a pronounced expansion of the functional detection range associated with early olfactory imprinting.

### Work endurance

Clear group differences were also observed for work endurance. Early-learning dogs maintained effective detection performance for extended periods, with a mean endurance time of 226.13 ± 6.54 min (*n* = 4), compared with 30.09 ± 0.30 min in control dogs (*n* = 10). All early-learning dogs exceeded the maximum endurance values recorded for control animals (Supplementary Table S3), suggesting a uniform enhancement of sustained task engagement rather than variability driven by isolated individual performance.

### Detection reliability

In contrast to the substantial differences observed for sensitivity, reaction distance, and endurance, detection reliability did not differ between groups. The false-positive index was comparable in early-learning dogs (1.92 ± 0.05, *n* = 4) and control dogs (1.90 ± 0.05, *n* = 20). Corresponding detection-rate values were 0.14 for the early-learning group and 0.15 for the control group (Supplementary Table S4). Individual values overlapped across groups, indicating that early olfactory imprinting did not modify false-positive responding or decision consistency under the conditions evaluated.

#### Summary of results

Taken together, the results show that early olfactory imprinting was associated with consistent and substantial improvements in detection sensitivity under tight-containment conditions, reaction distance, and work endurance. These effects were observed across all early-learning dogs and were not attributable to isolated individual performance. In contrast, detection reliability remained comparable between early-learning and conventionally trained dogs.

## Discussion

The present study provides evidence that early olfactory imprinting, when applied during the most plastic phases of development, is associated with substantial improvements in three core operational parameters: sensitivity in tight-containment conditions, reaction distance, and sustained work endurance. These findings are consistent with the classical notion of sensitive periods in behavioural and perceptual development, as described in foundational work on early learning and developmental plasticity (Wright and Russell 1983, Ortiz-Leal et al. 2020, Sarría-Echegaray et al. 2014).

The present findings can be interpreted through complementary mechanisms. From a classical-conditioning perspective, early repeated pairing of target odors with reinforcement is expected to increase the salience of those odors and facilitate subsequent discrimination learning; this is consistent with experimental work showing that odor pre-exposure and odor–reward pairing enhance acquisition and sensitivity in canine odor tasks (Hall et al. 2014, Hall et al. 2016). In parallel, very early exposure occurs during developmental stages characterized by heightened plasticity, and perinatal olfactory learning has been documented in domestic dogs (Battaglia 2009), while neonatal olfactory learning in mammalian systems has been associated with sensitive-period neurobiological mechanisms supporting durable behavioral effects (Beach 1963; Bertenthal 1987). Although neural measures were not collected here, the behavioral pattern is compatible with an interaction between associative learning and developmental timing.

The enhanced tightness-factor performance observed in early-learning dogs aligns with research showing that structured sensory exposure during early ontogeny facilitates more efficient discrimination thresholds and strengthens long-term perceptual stability. In our sample, dogs imprinted from very early stages displayed higher accuracy in conditions of minimal odour leakage, indicating an improved capacity to acquire, retain, and act upon weak olfactory cues. This pattern is compatible with the functional engagement of both the main olfactory system and accessory chemosensory pathways, particularly the vomeronasal organ, which is well developed in canids and contributes to the processing of complex odour stimuli.

The improvement in reaction distance is especially notable given the aerodynamic constraints associated with airborne odour dispersion. Previous studies have documented high detection accuracy in trained adult dogs operating under field-like conditions (Polgár et al. 2015). The markedly greater reaction distances shown by early-learning dogs in the present investigation suggest that early imprinting may shift operational detection thresholds, enabling earlier engagement with odour plumes and enhancing performance in open-area searches.

The results for work endurance also corroborate evidence that early sensory stimulation contributes to more resilient and stable behavioural profiles in adult dogs (Slabbert and Odendaal 1999). Early-learning dogs maintained operationally effective performance for substantially longer periods than control animals, suggesting that early associative routines may facilitate the development of motivational systems that support sustained attention, fatigue resistance, and emotional regulation, competencies that are essential in real operational contexts involving repeated searches and variable environmental demands.

In contrast, detection reliability, measured through false-positive behaviour and derived detection rates, did not differ significantly between groups. False positives depend not only on odour encoding but also on handler influence, reinforcement history, task structure, and the dog’s response strategy under uncertainty (Smith et al. 2003). The present findings indicate that early olfactory imprinting, although clearly beneficial for sensitivity, reaction distance, and endurance, does not independently modify these higher-order decision processes. This supports the view that response-inhibition mechanisms require targeted training at later developmental stages, when dogs possess the cognitive maturity to acquire more complex uncertainty-management rules.

From an applied perspective, the consistency of performance gains across independent variables suggests that early imprinting may help reduce the proportion of puppies that fail to meet operational standards in detection-dog programmes. Early stimulation and structured exposure may shape reactivity, self-control, and work motivation, variables associated with long-term operational suitability (Slabbert and Odendaal 1999). The similarity in performance between Hope and Vaiana, despite reduced exposure intensity and a later starting point for the latter, also suggests that early-learning protocols can be transferred across generations and adapted to different developmental contexts without loss of effectiveness.

This study has limitations, including the small number of dogs in the early-learning group and the quasi-experimental allocation inherent to the availability of operational animals. Nevertheless, the magnitude and internal consistency of the observed effects, together with the use of robust statistical approaches that account for unequal sample sizes and variances, support the reliability of the conclusions. The very large effect sizes found for tightness factor, reaction distance, and endurance reduce the likelihood that the results are attributable to chance or measurement artefacts.

Future studies should include larger samples, prospective designs with randomised allocation, and standardised testing environments to clarify the respective contributions of genetic predisposition, early exposure, and later training. Extending early imprinting to other scent categories, such as narcotics, human remains, or biological agents, may help determine the generality of these findings across detection disciplines. Further investigation is also warranted into how early learning interacts with advanced training protocols aimed specifically at reducing false positives, given that reliability remained comparable between groups in the present study.

Despite the consistency and magnitude of the observed effects, several limitations of the present study must be acknowledged. First, the early-learning group was necessarily small (*n* = 4), reflecting the operational and ethical constraints inherent to longitudinal work with detection dogs exposed to very early training protocols. This limits statistical power and precludes fine-grained modelling of inter-individual variability. Second, the study followed an observational, non-randomised design, as group allocation was determined by training history rather than experimental assignment. Although this reflects real-world operational conditions, it restricts causal inference. Third, part of the dataset relied on systematically recorded operational training logs rather than prospectively standardised experimental trials, which may introduce heterogeneity in measurement conditions despite the use of equivalent procedures across groups. Finally, the findings are specific to explosive detection and should not be automatically generalised to other detection disciplines (e.g., narcotics, biological agents or human remains) without further empirical validation. These limitations should be considered when interpreting the scope of the conclusions and highlight the need for future controlled studies with larger cohorts and broader detection contexts.

In summary, the findings demonstrate that early olfactory imprinting produces clear and operationally meaningful benefits in sensitivity, detection range, and sustained work endurance, while detection reliability remains unaffected. These results support the considered integration of early-learning protocols into breeding and training programmes for detection dogs, within controlled and evaluated operational frameworks.

## Conclusions

This study provides quantitative evidence that the timing of olfactory exposure plays a relevant role in shaping the long-term operational performance of detection dogs. Early olfactory imprinting was associated with large and consistent improvements across three independent performance domains, tight-containment detection, reaction distance, and sustained work endurance, indicating that early sensory exposure is linked to differences in the developmental expression of detection-related behaviours. These effects were not marginal: they involved substantial extensions in reaction range and pronounced gains in endurance, suggesting qualitative differences in functional performance rather than incremental improvements.

The absence of group differences in false-positive behaviour indicates that early imprinting selectively enhances the sensory and motivational foundations of detection work while leaving response-selection processes, such as inhibition and uncertainty management, unaffected. This distinction has practical relevance for training design, as early imprinting appears to optimise perceptual sensitivity and task engagement, whereas calibration of response control and decision thresholds likely requires targeted interventions at later stages of cognitive maturation.

The results further suggest that the effects of early imprinting are not confined to a single individual or lineage. Comparable performance profiles in dogs exposed at different developmental stages and with varying intensities of early exposure indicate that the protocol is robust across subjects and adaptable to different developmental contexts. Within the parameters assessed, early imprinting was associated with a shift in the overall performance profile, with potential implications for reducing attrition rates and enhancing baseline aptitude in detection-dog training programmes.

From a methodological perspective, this work demonstrates that early sensory interventions can be evaluated using operationally relevant performance metrics and robust statistical procedures. Although the number of dogs exposed to early imprinting was necessarily limited and the design observational, the magnitude and internal consistency of the effects observed across multiple, independent variables reduce the likelihood that the findings are attributable to random variation. Further controlled studies with larger cohorts and prospective designs will be required to clarify the relative contributions of genetic background, early exposure, and later training, and to determine the generalisability of these findings across other detection domains.

## Supplementary Information

Below is the link to the electronic supplementary material.

Supplementary File 1 (DOCX 15.0 KB)

## Data Availability

The datasets generated and analyzed during the current study are available from the corresponding author upon reasonable request.

## Declarations

### Statement on bioethics and animal welfare

This study strictly complies with ethical and legal principles regarding animal welfare. No invasive interventions, experimentation, physical manipulation, or procedures causing harm, stress, or suffering to the participating animals have been conducted. All activities were carried out in natural environments, respecting the developmental rhythms of the puppies and the usual training conditions in operational contexts. The Von Bormann protocol was designed as an early sensory stimulation methodology based on positive reinforcement, compatible with international recommendations on animal welfare in working animals. Consequently, evaluation by an animal ethics committee was not necessary, as it is not an experimental or clinical procedure, but an applied practice respectful of the animal’s nature and environment.
