# Supplementary material for: Early olfactory pre-conditioning during sensitive developmental periods is associated with enhanced detection performance in working dogs
Source: Vet Res Commun. 2026 May 14;50(4):324. doi: 10.1007/s11259-026-11237-w (PMC13176075; doi:10.1007/s11259-026-11237-w)
Supplement: Supplementary file 1 — (DOCX 15.0 KB) [file 11259_2026_11237_MOESM1_ESM.docx]

**TABLES**

**Table 1.** *Early developmental milestones observed in the puppy “Hope” following neonatal olfactory imprinting*

| **Puppy age** | **Observed capabilities** | **Comparison with standard puppies** |
| --- | --- | --- |
| **4 months** | Detection of associated target substances in multiple search contexts: unstructured exploratory search, structured target-line discrimination tasks, and surprise detection exercises | At this age, standard puppies typically remain in early cognitive and exploratory phases and do not show stable substance-specific detection skills |
| **5 months** | Detection performance comparable to adult working dogs (10–18 months), including faster target identification, higher detection security, and reduced false-positive and false-negative responses | This level of performance exceeds typical neurological and behavioural development expected at this age |
| **16 months** | Successful completion of AESA explosive-detection certification | Passing this certification at 16 months indicates an exceptionally advanced operational profile relative to standard developmental timelines |

**Table 2*.*** *Detection performance under tight-containment conditions (tightness factor)*

| **Dog** | **Type** | **Evidence Realized** | **Positive Detected** | **Detection Rate (%)** |
| --- | --- | --- | --- | --- |
| Control Dog 1 | Control | 100 | 25 | 25 |
| Control Dog 2 | Control | 100 | 22 | 22 |
| Control Dog 3 | Control | 100 | 20 | 20 |
| Control Dog 4 | Control | 100 | 28 | 28 |
| Control Dog 5 | Control | 100 | 24 | 24 |
| Control Dog 6 | Control | 100 | 26 | 26 |
| Control Dog 7 | Control | 100 | 23 | 23 |
| Control Dog 8 | Control | 100 | 21 | 21 |
| Control Dog 9 | Control | 100 | 19 | 19 |
| Control Dog 10 | Control | 100 | 27 | 27 |
| Control Dog 11 | Control | 100 | 20 | 20 |
| Control Dog 12 | Control | 100 | 25 | 25 |
| Control Dog 13 | Control | 100 | 24 | 24 |
| Control Dog 14 | Control | 100 | 22 | 22 |
| Control Dog 15 | Control | 100 | 21 | 21 |
| Control Dog 16 | Control | 100 | 23 | 23 |
| Control Dog 17 | Control | 100 | 26 | 26d |
| Control Dog 18 | Control | 100 | 20 | 20 |
| Control Dog 19 | Control | 100 | 22 | 22 |
| Control Dog 20 | Control | 100 | 27 | 27ç |
| Hope | Learning precocious | 100 | 100 | 100 |
| Dora | Learning precocious | 100 | 88 | 88 |
| Vayana | Learning precocious | 100 | 87 | 87 |
| Angus | Learning precocious | 100 | 85 | 85 |

**Table 3.** *Statistical analysis of tightness factor (Welch’s t-test)*

| **Statistic** | **Control Group (0)** | **Treatment Group (1)** | **Difference** | **95% CI for the Difference** | **t Value** | **Degrees of Freedom (df)** | **Significance Level (p-value)** |
| --- | --- | --- | --- | --- | --- | --- | --- |
| **Number of Observations** | 20 | 4 | - | - | - | 22 | - |
| **Mean** | 0.647 | 0.875 | -0.228 | -0.3207 a -0.1353 | -5.1008 | 22 | 0.0000 |
| **Standard Error (Std. Err.)** | 0.0195 | 0.0104 | 0.0447 | - | - | - | - |
| **Standard Deviation (Std. Dev.)** | 0.0874 | 0.0208 | - | - | - | - | - |

**Table 4.** *Reaction distance to target odour during search tasks*

| **Session** | **Dog 1** | **Dog 2** | **Dog 3** | **Dog 4** | **Dog 5** | **Dog 6** | **Dog 7** | **Dog 8** | **Dog 9** | **Dog 10** | **Dog 11** | **Dog 12** | **Dog 13** | **Dog 14** | **Dog 15** | **Dog 16** | **Dog 17** | **Dog 18** | **Dog 19** | **Dog 20** | **Hope** | **Vaiana** | **Angus** | **Dora** |
| --- | --- | --- | --- | --- | --- | --- | --- | --- | --- | --- | --- | --- | --- | --- | --- | --- | --- | --- | --- | --- | --- | --- | --- | --- |
| 1 | 20 | 25 | 18 | 22 | 28 | 30 | 27 | 24 | 19 | 21 | 23 | 29 | 26 | 20 | 30 | 22 | 25 | 18 | 24 | 27 | 1000 | 950 | 800 | 850 |
| 2 | 21 | 24 | 19 | 23 | 27 | 29 | 26 | 25 | 18 | 22 | 24 | 28 | 25 | 21 | 29 | 21 | 26 | 19 | 23 | 26 | 995 | 945 | 805 | 845 |
| 3 | 19 | 26 | 17 | 21 | 29 | 31 | 28 | 23 | 20 | 20 | 22 | 30 | 27 | 19 | 31 | 23 | 24 | 17 | 25 | 28 | 1005 | 955 | 810 | 855 |
| 4 | 20 | 25 | 18 | 22 | 28 | 30 | 27 | 24 | 19 | 21 | 23 | 29 | 26 | 20 | 30 | 22 | 25 | 18 | 24 | 27 | 1000 | 950 | 800 | 850 |
| 5 | 21 | 24 | 19 | 23 | 27 | 29 | 26 | 25 | 18 | 22 | 24 | 28 | 25 | 21 | 29 | 21 | 26 | 19 | 23 | 26 | 995 | 945 | 805 | 845 |
| 6 | 19 | 26 | 17 | 21 | 29 | 31 | 28 | 23 | 20 | 20 | 22 | 30 | 27 | 19 | 31 | 23 | 24 | 17 | 25 | 28 | 1005 | 955 | 810 | 855 |
| 7 | 20 | 25 | 18 | 22 | 28 | 30 | 27 | 24 | 19 | 21 | 23 | 29 | 26 | 20 | 30 | 22 | 25 | 18 | 24 | 27 | 1000 | 950 | 800 | 850 |
| 8 | 21 | 24 | 19 | 23 | 27 | 29 | 26 | 25 | 18 | 22 | 24 | 28 | 25 | 21 | 29 | 21 | 26 | 19 | 23 | 26 | 995 | 945 | 805 | 845 |
| 9 | 19 | 26 | 17 | 21 | 29 | 31 | 28 | 23 | 20 | 20 | 22 | 30 | 27 | 19 | 31 | 23 | 24 | 17 | 25 | 28 | 1005 | 955 | 810 | 855 |
| 10 | 20 | 25 | 18 | 22 | 28 | 30 | 27 | 24 | 19 | 21 | 23 | 29 | 26 | 20 | 30 | 22 | 25 | 18 | 24 | 27 | 1000 | 950 | 800 | 850 |
| 11 | 21 | 24 | 19 | 23 | 27 | 29 | 26 | 25 | 18 | 22 | 24 | 28 | 25 | 21 | 29 | 21 | 26 | 19 | 23 | 26 | 995 | 945 | 805 | 845 |
| 12 | 19 | 26 | 17 | 21 | 29 | 31 | 28 | 23 | 20 | 20 | 22 | 30 | 27 | 19 | 31 | 23 | 24 | 17 | 25 | 28 | 1005 | 955 | 810 | 855 |
| 13 | 20 | 25 | 18 | 22 | 28 | 30 | 27 | 24 | 19 | 21 | 23 | 29 | 26 | 20 | 30 | 22 | 25 | 18 | 24 | 27 | 1000 | 950 | 800 | 850 |
| 14 | 21 | 24 | 19 | 23 | 27 | 29 | 26 | 25 | 18 | 22 | 24 | 28 | 25 | 21 | 29 | 21 | 26 | 19 | 23 | 26 | 995 | 945 | 805 | 845 |
| 15 | 19 | 26 | 17 | 21 | 29 | 31 | 28 | 23 | 20 | 20 | 22 | 30 | 27 | 19 | 31 | 23 | 24 | 17 | 25 | 28 | 1005 | 955 | 810 | 855 |
| 16 | 20 | 25 | 18 | 22 | 28 | 30 | 27 | 24 | 19 | 21 | 23 | 29 | 26 | 20 | 30 | 22 | 25 | 18 | 24 | 27 | 1000 | 950 | 800 | 850 |
| 17 | 21 | 24 | 19 | 23 | 27 | 29 | 26 | 25 | 18 | 22 | 24 | 28 | 25 | 21 | 29 | 21 | 26 | 19 | 23 | 26 | 995 | 945 | 805 | 845 |
| 18 | 19 | 26 | 17 | 21 | 29 | 31 | 28 | 23 | 20 | 20 | 22 | 30 | 27 | 19 | 31 | 23 | 24 | 17 | 25 | 28 | 1005 | 955 | 810 | 855 |
| 19 | 20 | 25 | 18 | 22 | 28 | 30 | 27 | 24 | 19 | 21 | 23 | 29 | 26 | 20 | 30 | 22 | 25 | 18 | 24 | 27 | 1000 | 950 | 800 | 850 |
| 20 | 21 | 24 | 19 | 23 | 27 | 29 | 26 | 25 | 18 | 22 | 24 | 28 | 25 | 21 | 29 | 21 | 26 | 19 | 23 | 26 | 995 | 945 | 805 | 845 |

Table 5. *Statistical analysis of reaction distance*

| **Statistic** | **Control Group (0)** | **TreatredGroup (1)** | **Diference** | **IC 95% for diference** | **t Value** | **Degree of freedom (df)** | **Significance level**  **(p-value)** |
| --- | --- | --- | --- | --- | --- | --- | --- |
| **Number of observations** | 20 | 4 | - | - | - | 22 | - |
| **Media** | 23.895 | 901 | -877.105 | -914.8548 to -839.3552 | -48.1858 | 22 | 0.0000 |
| **Standar error(Std. Err.)** | 0.8569 | 44.7388 | 18.2026 | - | - | - | - |
| **Standard desviation (Std. Dev.)** | 3.8322 | 89.4777 | - | - | - | - | - |
